# Sirtuins are Not Conserved Longevity Genes

**DOI:** 10.1093/lifemeta/loac025

**Published:** 2022-09-22

**Authors:** Charles Brenner

**Affiliations:** Department of Diabetes & Cancer Metabolism, Beckman Research Institute of City of Hope, Duarte, CA 91010 USA

## Abstract

It is central to biology that sequence conservation suggests functional conservation. Animal longevity is an emergent property of selected traits that integrates capacities to perform physical and mental functions after reproductive maturity. Though the yeast *SIR2* gene was nominated as a longevity gene based on extended replicative longevity of old mother cells, this is not a selected trait: *SIR2* is selected against in chronological aging and the direct targets of *SIR2* in replicative lifespan are not conserved. Though it would be difficult to imagine how a gene that advantages 1 in 5 million yeast cells could have anticipated causes of aging in animals, overexpression of *SIR2* homologs was tested in invertebrates for longevity. Because artifactual positive results were reported years before they were sorted out and because it was not known that *SIR2* functions as a pro-aging gene in yeast chronological aging and in flies subject to amino acid deprivation, a global pursuit of longevity phenotypes was driven by a mixture of framing bias, confirmation bias and hype. Review articles that propagate these biases are so rampant that few investigators have considered how weak the case ever was for sirtuins as longevity genes. Acknowledging that a few positive associations between sirtuins and longevity have been identified after thousands of person-years and billions of dollars of effort, we review the data and suggest rejection of the notions that sirtuins 1) have any specific connection to lifespan in animals and 2) are primary mediators of the beneficial effects of NAD repletion.

## What Constitutes a Conserved Gene

Biology became a molecular science in the 20^th^ century with the convergence of classical genetics, the central dogma of molecular biology, and biochemical advances^1^. As I explain to my students, we understand that things are the way they are in biology on account of two rules:

Rule 1) Biology does not violate of any rule of chemistry or physics.
Rule 2) Biological traits are encoded by nucleic acids and passed down by mutation and selection.

Rule 1 compels us away from fantastical ideas like perpetual motion and time-independent processes. Rule 2 tells us that functions are constrained by inherited macromolecular sequences.

As a central principle of biology, Rule 2 allows us to define a conserved gene as a nucleotide or amino acid sequence under selective pressure to do some specific function. If we take the example of hexokinase, the function is phosphorylation of the 6 oxygen of glucose using ATP as the phosphoryl donor. If potential hexokinase sequences are identified on the basis of sequence similarity, we can test whether such sequences encode a hexokinase. Hexokinase sequences could drift into and be selected for additional or consider them to be conserved hexokinase orthologs if they still fulfill the function of hexokinase but we’d call them paralogs if they are related by descent but are doing something else. A hexokinase might also have an emergent property such as promoting short or long lifespan in one particular life form. While one might postulate that this function is conserved throughout evolution, this thesis would have to hold up to experimental testing in order to be warranted. Indeed, we can expect that any enzyme family might have a conserved biochemical activity and distinct functional consequences revealed by mutation in different forms of life that are not conserved. Both things can be true at the same time.

## Sirtuins are Conserved as NAD-Dependent Protein Lysine Deacylases

**Fig. 1.**
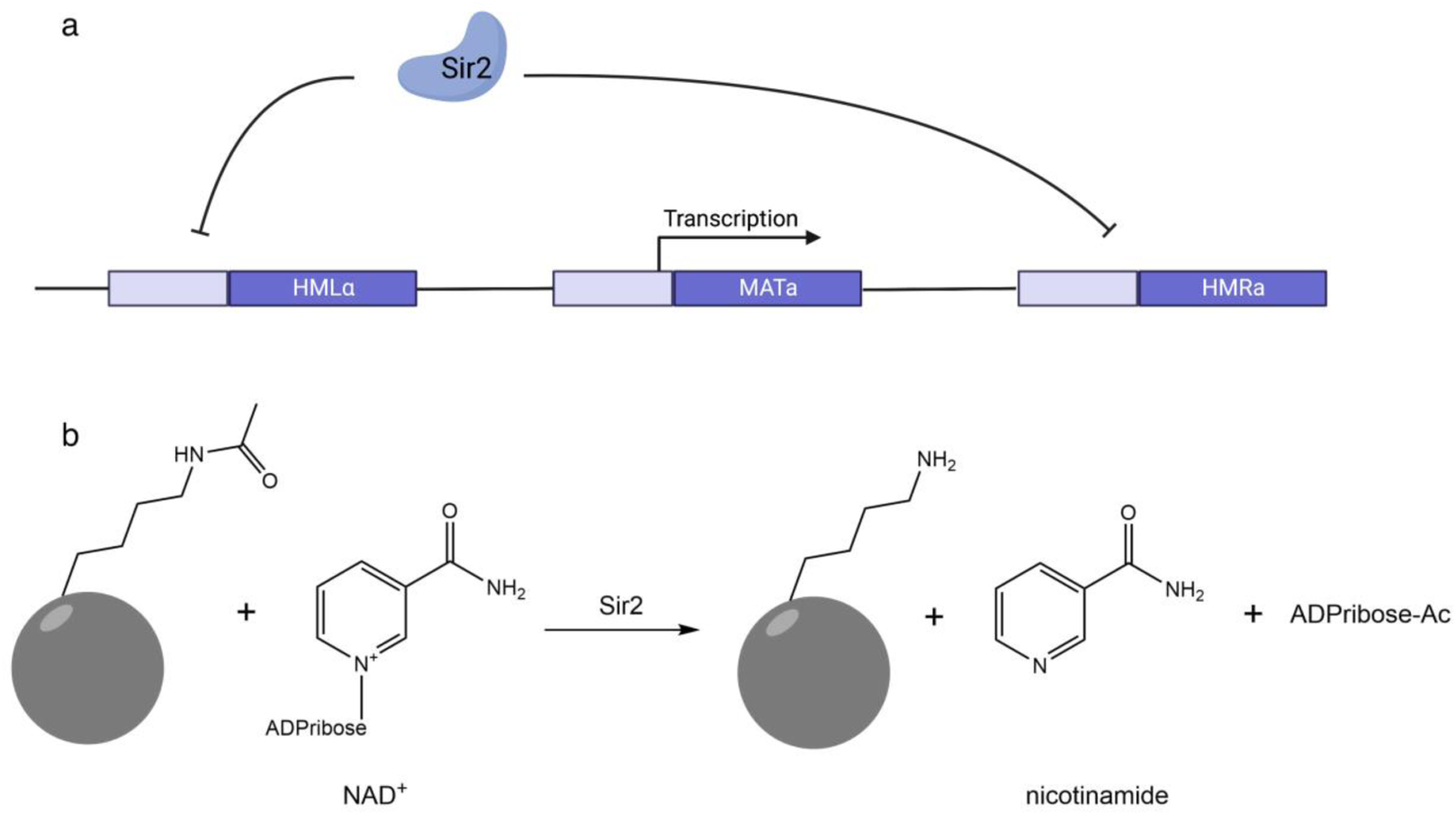
Sir2 Enzymes are Gene Silencers in Fungi and are Conserved as NAD^+^-Dependent Protein Lysine Deacylases **a.** In S. *cerevisiae*, the *SIR2* gene is required to silence genes in particular chromosomal locations. This function is to be conserved across divergent yeasts. **b.** The biochemical function of sirtuins is largely conserved. These enzymes deacylated protein lysine substrates in a manner that depends on NAD^+^ and which produces the deacylated protein lysine substrate, nicotinamide and acylated ADPribose.

The budding yeast *Saccharomyces cerevisiae* has haploid and diploid phases of its life cycle. Haploids come in two mating types, termed **a** and α and are capable of undergoing a zygotic process to form **a**/α diploids. Like haploids, diploids divide vegetatively, and they can also sporulate to form two **a** and two α haploids. Peculiarly, haploid yeast express **a** or α mating type information from the *MAT* locus but have cryptic copies of mating type genes on either side of *MAT* on chromosome 3. The system exists so that haploids can switch mating type in order to mate and form diploids. On account of the additional copies of mating type information, yeast has a system to keep the cryptic copies silenced^2^. The *SIR2* gene is one of four genes required to keep cryptic mating type information silent (Fig. 1a)^3–5^. In other budding yeasts, such as *Candida albicans*, loss of *SIR2* leads to a high rate of phenotypic switching, potentially through a gene silencing mechanism^6^. In the fission yeast *Schizosaccharomyces pombe*, the SIR2 homolog clearly has a conserved function in gene silencing^7^, suggesting that *SIR2*-homologous genes were conserved throughout disparate fungal lineages for gene silencing.

Biochemically, the Sir2 protein was shown to remove acetyl modifications of protein Lys sidechains in a manner that depends on nicotinamide adenine dinucleotide (NAD^+^)^8–10^. This activity has exhibited remarkably little drift across Sir2-homologous enzymes, which are termed sirtuins; nearly all of these enzymes remove an acetyl or other acyl modification of lysine in a manner that liberates nicotinamide (NAM) and which links the acyl group to thw 2’ and 3’ oxygens on the leaving group ribose^11^ (Fig. 1b). Clearly, this biochemical activity of sirtuins—found in bacteria, archaea, plants, animals and fungi^12^–has been highly conserved. In sum, it is not controversial that sirtuins have an exceptionally well conserved biochemical function and that, in yeasts, they have a conserved function in gene silencing.

## Two Models of Aging in *S. cerevisiae* Disprove the Notion that SIR2 was Conserved as the Mediator of the Longevity Benefit of Caloric Restriction

The nonsexual component of the *Saccharomyces cerevisiae* lifecycle is characterized by budding. Yeast cells replicate until they have used up essential inputs. Yeast lifespan can be characterized in two completely different ways termed replicative aging and chronological aging, both of which are extended by caloric restriction (CR)^13^. Despite the fact that CR extends lifespan in both models, *SIR2* advantages old mothers in the replicative aging model^14^ while disadvantaging all cells in the chronological aging model^15^. Thus, it cannot be said that *SIR2* is conserved as a mediator of the longevity benefit of CR even within *Saccharomyces cerevisiae*.

**Fig. 2.**
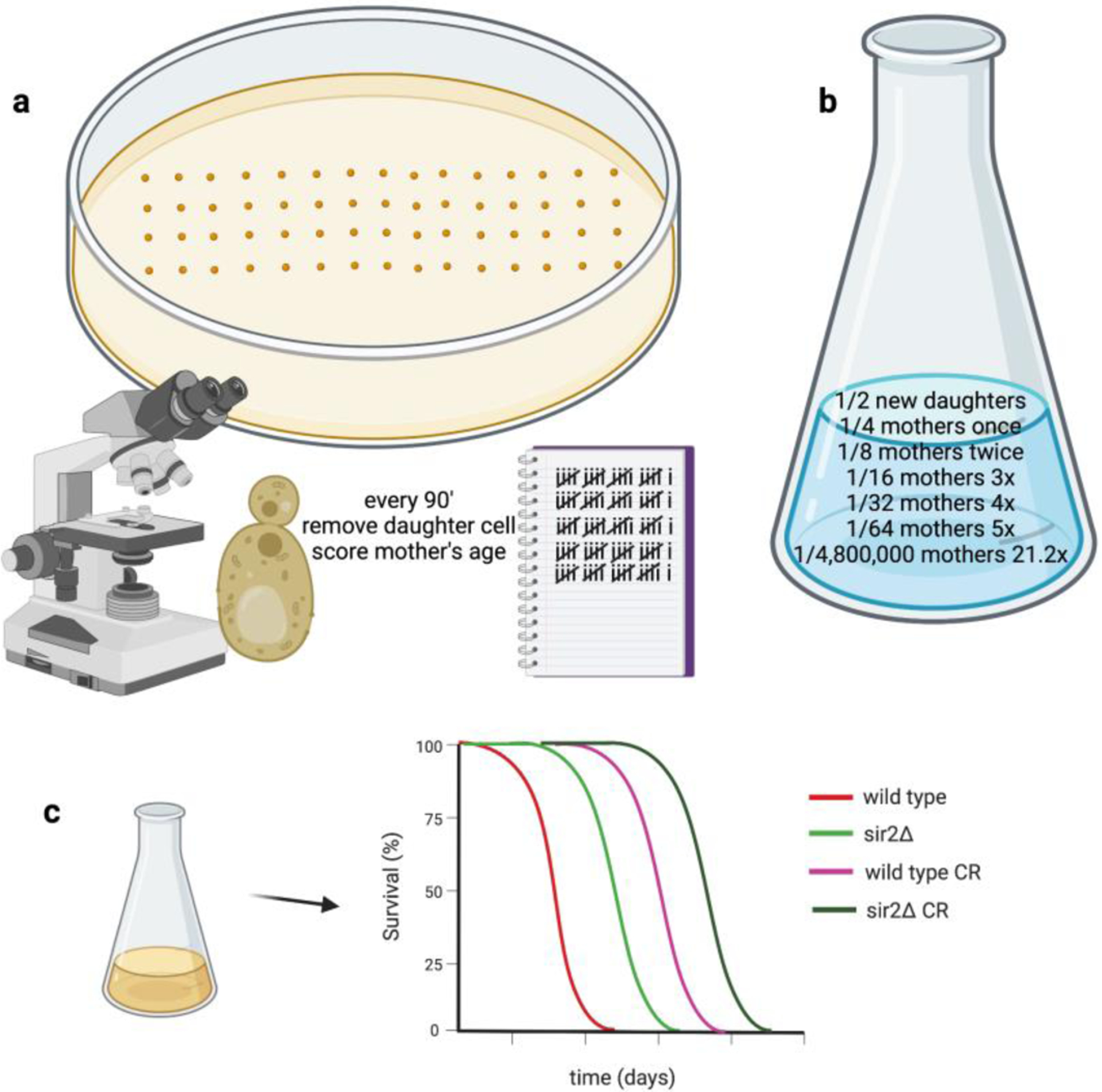
Sir2 favors a nonselected and disfavors a selected type of aging in budding yeast, both of which are extended by calorie restriction **a.** In replicative aging, cells are arrayed on a petri dish for a two-week experiment in which daughter cells are removed every time the mother cell has replicated. The longevity benefit of CR—and the effect of *SIR2*—emerges after about 21.2 cell divisions^16^. **b.** In a yeast culture mothers who have divided 21.2 times constitute 1 in 4.8 million cells. Because a yeast culture can be regenerated from any cell, this is not a selected trait. **c.** Yeast have been recultured from the bottom of flasks, bottles and pottery for millenia: the ability to regrow a culture over an extended period of time after the original culture exhausted resources is a selected trait termed chronological lifespan. CR extends chronological lifespan in yeast and does so better with *sir2* deletion^15^.

Existing cells are termed mothers while the buds they form are termed daughters. In the replicative lifespan assay, new daughters are arrayed on plates to serve as mothers. Every 90 minutes or so, laboratory workers remove the daughters to score how many times a single mother cell can produce daughters—this process continues for approximately 2 weeks until exhausted old mothers can no longer produce daughters (Fig. 2a).

It is essential to note that in a dividing culture, half of the cells are new daughters, who have never been mothers, and half of the mothers have only been mothers one time. Thus, old mothers are rare cells with a calculated frequency of ½^(n + 1)^ where n is the number of times a mother cell has had daughters (Fig. 2b). The actual frequency of n-time mothers is, in fact, lower because mothers lose proficiency at forming daughters in their old age. In high glucose conditions, the average yeast mother lifespan is 21.2 generations, whereas in CR conditions, the average yeast mother lifespan is 26.2 generations^16^, meaning that CR allows a mother that comprises only 1 in 5 million cells (1/2^22.2^ = 1/4.8 × 10^6^) to divide another 5 times. The longevity benefit of CR in this model depends on *SIR2*^16^ and has been attributed to accumulation of extrachromosomal ribosomal DNA circles (ERCs) in the yeast mother cells^14^. However, as a yeast culture can be regrown from any single cell, the exceedingly rare old mother cells are not under selective pressure to retain *SIR2* function. In addition, ERCs are not conserved as either a *SIR2* target or a cause of aging in other organisms and the centrality of *SIR2* as the mediator of the replicative longevity benefit of CR has been strongly questioned with respect to the effect of strain backgrounds^17^. Thus, the ability of *SIR2* to repress formation of ERCs can be defined as a nonconserved function of sirtuins that advantages rare old mother cells, allowing them a few additional cell divisions when grown in CR conditions.

The way Rule 2 works, of course, is that a trait must be under selective pressure to constrain macromolecular sequences and functions. Because a yeast culture can be regenerated from any one cell, there simply is no selective pressure on the *SIR2* gene in one highly dispensable cell out of 5 million to divide five more times.

While the replicative aging model provides no fitness advantage, the chronological aging model does provide clear selective advantages. In this model, a culture is grown to stationary phase and then plated over the next few days and weeks to score cell survival by the ability to form a colony. Unlike the replicative aging model in which the viability of the culture is unaffected by whether the oldest mothers continue to divide, chronological aging resembles every storage and survival condition that yeast has faced since it was first cultivated thousands of years ago. For yeast to be able to support the next batch of wine, bread or beer, it has to remain viable when it has run out of nutrients and stored. CR extends chronological lifespan but the presence of a wild-type *SIR2* gene shortens lifespan and greatly limits the lifespan extension that is experienced on CR^15^ (Fig. 2C).

Based on these data, it is logical to conclude that if yeast had a strong selection for lifespan, the *SIR2* gene would have been lost^15^ and thus, its presence in the yeast genome suggests it has been conserved for other functions, potentially related to gene silencing^3,4,6,7^. Given the fact that the replicative longevity phenotype only advantages 1 in 5 million cells^16^ via a mechanism and targets that are not conserved in the aging of other organisms^14^, it would be unreasonable to nominate *SIR2* as a conserved central controller of aging in animals. Unfortunately, the disadvantage conferred by *SIR2* in the evolutionarily selected type of aging was not described until 2005^15^ and this was long after dozens of review articles and pieces in the popular press had celebrated the fantastical idea that a yeast gene anticipated a limiting factor in animal aging. A small fraction of such reviews is here referenced^18–21^.

## The Evolutionary Basis for Aging in Animals

Animals emerged from a last common eukaryotic ancestor approximately 600 M years ago—the emergence of animal-like sexual mating appears to have been earlier than this^22^. The complexity of heterotrophic and sexually dimorphic animal life demands that individuals are able to acquire food, avoid predation, develop to reproductive maturity, mate, and protect their offspring until their offspring are capable of all of these functions^23,24^.

While animals that can only reproduce once are under no selective pressure to continue to live beyond their contribution to the gene pool, most animals evolved with the capacity to undergo multiple cycles of reproduction. From this capability, animal longevity appears to be an emergent property of all of the fitness traits that enable creatures to acquire resources, mate and promote the success of their offspring^24^. What have been termed “Hamilton’s forces of natural selection”^25^ make animals strong, clever, sex-appealing, fecund and protective of their young^23,24^. To the degree that animals spread their genes by repeated cycles of mating and avoid predation and disease, they can also be long-lived with respect to the onset of their reproductive maturity. There are solid observations that the longest lived animals experience little predation, have a late onset of reproductive maturity, and remain reproductively active throughout life^26,27^. Human females are an important exception to this rule because they experience menopause at midlife: postreproductive female health is evidence for the important roles of mothers and grandmothers in the survival and reproductive success of young people^28^.

Realizing that inbred male and female flies that are exposed to each other from early life are under little selective pressure for longevity, flies were kept as virgin males and females until late life in order to select for postponed senescence^29^. Genomic analysis of the long lived flies that were obtained from these selections support the proposition that the genetic determinants of animal longevity are highly polygenic^30^. Consistent with theory and experiment, analysis of the genomes of long-lived humans has not revealed strong monogenic longevity genes but rather complex webs of gene by environment and gene by gene interactions that allow some people to retain their faculties into a second century^31^.

In animal models, there have been a few notable discoveries of monogenic longevity mutants, which almost invariably map to loss of function alleles of genes under selection for vital processes such as growth and reproduction. For example, *Mth* encodes a developmentally essential G-protein coupled receptor (GPCR) in flies, deletion of one copy of which extends lifespan^32^ at the expense of neuromuscular function^33^. Though the GPCR superfamily is conserved across all biological kingdoms, *Mth* is apparently under selective pressure for development and neuromuscular function and it does not have vertebrate orthologs^34^.

The largest class of monogenic longevity mutants map to insulin-like growth factor (IGF-1) and growth hormone signaling pathways that are conserved between worms and mammals. These include the developmentally essential *daf-2* gene in worms^35,36^ and the genes associated with long-lived dwarf mice including those encoding and/or supporting production of growth hormone, IGF-1, prolactin, thyroid-stimulating hormone, and growth hormone receptor^37^. Dwarf mice are stunted in growth, unable to compete for resources with wild-type littermates, cold-sensitive and infertile. Thus, while in the laboratory, monogenic reduction in pituitary signaling clearly extends lifespan, it is equally clear that the genes were conserved to promote growth and development. Further, for reasons that are not understood but are almost certainly due to the many differences between free-living humans and laboratory mice, human dwarf syndromes linked to the same genes do not produce long-lived people^38^.

In sum, because animal longevity integrates the function of all vital organ systems over time, it is highly polygenic. Monogenic regulators of mammalian longevity clearly exist, though the genes have been conserved for growth and development^38^.

## Highly Cited, Nonreproducible Experiments Established the Dogma that Invertebrate Sirtuins Extend Lifespan in Animals

2001 was a heady time in which the first assemblies of the human genome were published^39^ and two-thirds of the Physiology Nobel Prize for discovery of key regulators of the cell cycle was awarded for work in yeast by Lee Hartwell and Paul Nurse^40^. When the ERC-repressing mechanism for yeast replicative lifespan extension was published four years prior, the extremely low abundance of the target cell was clearly discussed^14^. However, this discussion was not included in more recent work, which claimed centrality for *SIR2* in mediating the longevity benefit of CR in yeast^16^. More thorough work refuting this had not yet been published^17^ and the selective disadvantage conferred by *SIR2* in chronological aging remained undiscovered^15^. It therefore must have seemed reasonable to test whether additional copies of the worm and fly *SIR2* orthologs might extend lifespan in invertebrate model organisms.

Indeed, it was reported in Nature that an extra copy of *sir-2.1* extended worm lifespan by “up to 50%”^41^. Accompanying this paper, David Gems opined “arguably, using budding yeast (*Saccharomyces cerevisiae*) to investigate the genetic determinants of ageing, and consequently longevity, seems almost absurdly optimistic. Ageing yeast do not develop grey hair or poor eyesight, or start complaining about young people today, or have strokes. In fact, it is not even clear that they age at all, and when researchers talk of yeast ‘lifespan’, what they really mean is the number of times a yeast mother cell can reproduce by producing a bud”^42^. After examining the data showing apparent worm lifespan extension by an extra copy of *sir-2.1*^41^, Gems considered the result plausible and wrote that “it seems that some genetic determinants of longevity and ageing are conserved across animal groups”^42^. Shortly thereafter, overexpression of the apparent *Drosophia* ortholog of *SIR2* was reported to extend lifespan in flies by mediating the effect of CR^43^.

Model building and storytelling are key parts of science. Proponents of the centrality of *SIR2* as a mediator of the longevity benefit of CR told a compelling story. If you ignored the follow-up work on the dispensability of *SIR2* for CR-extended longevity^17^ and did not consider the problem of the total dispensability of 1 in 5 million cells^14^, you could focus on the attractive idea that CR extends lifespan by virtue of beneficial stress that links metabolism to youthful gene expression^16^. Proponents of this model told us in multiple review articles that even though the targets of *SIR2* are not conserved from one organism to another, they are conserved for the purpose of longevity extension^18–20,44^. With the apparent *SIR2* life extension results in worms^41^ and flies^43^ that seemed to support *SIR2* homologs as dominantly acting longevity genes, the strain-specific effects in yeast seemed to be neutralized^17^.

Companies were formed, activators were sought, mouse models were created, review articles were written, grants were funded, and laboratories globally were mobilized to better understand human *SIRT1*, which was described as the *SIR2* ortholog among a “magnificent” set of seven sirtuins that would revolutionize human medicine^45^. The amount of global hype around a yeast gene said to be conserved as a family of dominantly acting animal longevity genes is difficult to overstate. Indeed, this hype has permeated popular culture in books, podcasts and social media^46^. Moreover, this author agrees with David Gems that if a yeast gene were to have anticipated the limiting factors in animal aging, the unbridled enthusiasm about sirtuins would be warranted.

A decade after the initial worm result, Gems was the senior author of a paper written by investigators from seven different institutions, who wrote that “in *C. elegans*, outcrossing of a line with high level *sir-2.1* over-expression abrogated the longevity increase, but not *sir-2.1* over-expression. Instead, longevity co-segregated with a second-site mutation affecting sensory neurons. Outcrossing of a line with low copy number *sir-2.1* over-expression also abrogated longevity. A *Drosophila* strain with ubiquitous over-expression of dSir2 using the UAS-GAL4 system was long-lived relative to wild-type controls, as previously reported, but not relative to the appropriate transgenic controls, and nor was a new line with stronger overexpression of dSir2. These findings underscore the importance of controlling for genetic background and the mutagenic effects of transgene insertions in studies of genetic effects on lifespan. The life extending effect of dietary restriction on ageing in *Drosophila* has also been reported to be dSir2 dependent. We found that dietary restriction increased fly lifespan independently of dSir2”^47^.

There continued to be some back and forth with respect to overexpression of *SIR2* homologs in invertebrates. Acknowledging strain artifacts, the original proponents pared back and attempted to defend claims of lifespan extension in worms^48^ and flies^49^. After all the controls were carefully performed, it is now clear that the worm longevity effect of *sir-2.1* is not only strain-specific but also dependent on inclusion of thymidylate synthase inhibitor 5-fluorodeoxyuridine to block development of progeny^50^. In flies, independent laboratories reported that loss of dSir2 does not shorten lifespan^51,52^ and that, in fact, deletion of one copy of this gene extends lifespan and greatly extends lifespan when flies are starved for amino acids^52^. There is not a general reproducibility problem with these assays because multiple methods of *daf-2* inhibition are reproducible as lifespan extenders in worms^53^, whereas worm and fly Sir2 overexpressers are not and, in fact, dSir2 antagonizes healthy aging in the context of amino acid restriction^52^.

The problem we face is that the thesis that not just one but seven sirtuins function as dominantly acting longevity genes has been canonized in major lectures such as the Franklin H. Epstein Lecture in the New England Journal of Medicine^54^. There are many hundreds of highly cited review articles on sirtuins and aging, which are premised on sirtuins conserved in yeast as longevity genes, sirtuins conserved in invertebrates in longevity genes, and sirtuins functioning as mammalian longevity genes. These reviews refer to each other for support and make little effort to question the underlying experimental support for the thesis. Indeed, the persistent influence of review articles and media pieces that are premised on overinterpreted or irreproducible data are such that this author was asked by dozens of scientists, multiple journal editors, and hundreds of laypeople to put together this critique.

## The Story of Resveratrol as a Sirtuin Activator Constitutes a Deep Contamination of the Scientific Literature

Resveratrol is a polyphenolic compound found in peanuts and red wine that had already been the subject of a great deal of speculation prior to 2000^55^. The French Paradox—a low incidence of coronary heart disease despite a high consumption of saturated fat—had been correlated with red wine consumption^56^ so that people were motivated to identify health-promoting compounds in wine. Notably, there are countless potential explanations of the French Paradox, such that there is little likelihood that any single factor explains it and no limit to the stories that are created to support one’s favorite cardioprotective food group or lifestyle^57^.

**Fig. 3.**
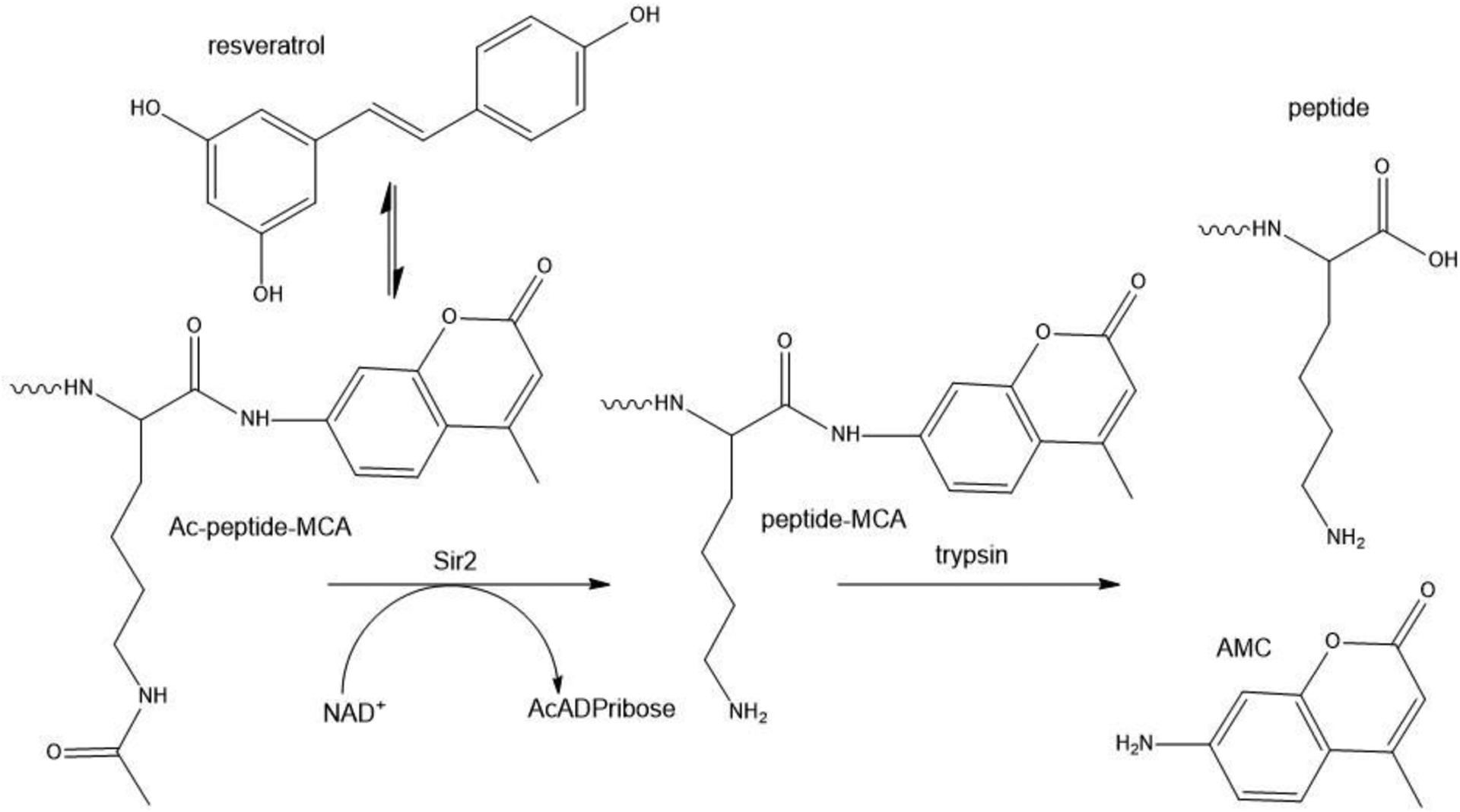
The fluorophore in peptide substrates used to screen for sirtuin activators interacts with resveratrol and other proposed activators Sir2 was screened for activators using an acetylated peptide-methylcoumarinamide substrate in which the deacetylated product was incubated with trypsin to release aminomethylcoumarin, which is fluorigenic^58^. Resveratrol and other activators interact with the reporter group^64,65,72^.

Using a synthetic peptide substrate containing an aminomethylcoumarin reporter group, several polyphenols including resveratrol, fisetin and quercetin were reported to increase the activity of human Sirt1 (Fig. 3A). In the same paper, resveratrol was reported to extend the lifespan of yeast in the replicative aging model by functioning as a Sir2 activator and CR mimetic^58^. The investigators postulated the “xenohormetic” concept that fungal and animal sirtuins are activated by compounds produced when plants in their environment are stressed. The Nature paper was accompanied by a commentary entitled “Ageing: a toast to long life”^59^ and coverage in Science entitled “Longevity research: In vino vitalis? Compounds activate life-extending genes”^60^. Despite as many as 20 thought pieces on xenohormesis^61–63^, the idea has never been tested (*i.e.*, would extracts of stressed versus nonstressed plants extend the life of insects?) but has entered global consciousness through a bestselling book and podcasts as though it were a scientifically established fact^46^.

To publish a paper in a biochemistry specialist journal in which one identifies a putative enzyme activator or inhibitor, reviewers expect to see evidence that the small molecule-enzyme interaction is direct and is not artifactually related to the screening method. Such validating work was not apparently requested in the definition of resveratrol as a xenohormetic lifespan-extending Sirt1 activator^58^. As the Sirt1 peptide substrate contained aminomethylcoumarin and also required trypsin to generate a signal, this was a clear failure of peer review. It was soon discovered that resveratrol does not activate Sirt1-dependent deacetylation of peptide substrates without the fluorescent reporter group^64,65^. Further, in contrast to the initial report, resveratrol fails to extend lifespan of yeast or influence Sir2 activity in vivo^17^. Resveratrol was reported to extend lifespan in flies and worms by activating Sir2 homologs^66^ but this effect could not be independently replicated^67^.

How could this happen? Yeast replicative aging assays are done on plates that are kept for about two weeks time and are sensitive to humidity and other factors that are hard to control. If researchers are not careful, they could fool themselves into thinking that particular genetic or pharmacological interventions have an effect when the effects are driven by humidity or noise. Worse yet, researchers could throw out results that they cannot explain in order to construct a story in which all the pieces (representing noise) come together in a pleasing way. In their attempt to replicate the reported longevity-promoting effects of resveratrol in worms, the variability of the assays were noted^67^—it is therefore critical that data are not selected in order to be consistent with a story. It is also unfortunate that laboratory investigations are not generally performed in a blinded manner: laboratory personnel generally open-label their experiments and can be unduly influenced by their preconceived notion of what experiments are working versus what experiments can be discarded because they are not producing the expected results. Further, in large laboratories, there are cases in which more than one person is trying to obtain a result that the lab director has publicly predicted. This can lead to situations in which beautiful noise is selected and independent replication will fail.

Resveratrol is widely known to be a dirty drug with dozens of cellular targets and mechanisms^68^. When resveratrol was added to a high fat diet, mice gained somewhat less weight, generally outperformed mice on the high fat diet, and had a higher density of mitochondria in liver^69^. By Occam’s razor, one might assume that resveratrol depressed fat absorption by any number of mechanisms, such that more fat was eliminated in the treated animals than the nontreated. However, the caloric content of fecal matter was not measured. The researchers did not conduct any direct or genetic test for Sirt1 activation but rather performed a western blot for the acetylation state of liver PGC1α as a proxy for Sirt1 activity and reported that resveratrol-treated animals had a lower acetylation state than in the nontreated high fat animals^69^. In fasting, PGC1α deacetylation had been linked to Sirt1 activity and mitochondrial biogenesis^70^ so it sort of made sense that Sirt1 might have acted on PGC1α in the high fat-fed liver. However, we know that resveratrol acts through multiple mechanisms and the beneficial effect of resveratrol was only seen on high fat, in contrast to the expectations that Sirt1 would be more important in CR conditions. Moreover, there are dozens of factors that would influence the acetylation status of PGC1α in liver that were not examined including determination of the percent of hepatocytes versus inflammatory cells, and determining the activity of all of the potential acetyltransferases and deacetylases that act on PGC1α. Thus, irrespective of what global announcements claimed the paper showed, the investigators surely did not provide any evidence that resveratrol causes Sirt1 to respond as though mice were in CR^69^.

Nature accompanied the article with an piece entitled, “Let them eat cake.” The editorial read “You can have your cake and eat it. Fat, healthy and tipsy. Fountain of youth. These headlines and more greeted online publication of the article ‘Resveratrol improves health and survival of mice on a high-calorie diet’. What the paper does show is that consumption of resveratrol at doses achievable in humans (but not from red wine — the hundreds of bottles a day needed would have side effects) can reproduce many of the physiological effects of a low-calorie diet in mice, improving health and survival.” A selection of New York Times articles from this era were entitled “Fighting the effects of fat: Pass the pinot (Nov 1, 2006),” “Yes, Red Wine Holds Answer. Check Dosage (Nov 2, 2006),” “Aging Drugs: Hardest Test Is Still Ahead (Nov 7, 2006),” “An Age-Defying Quest (Red Wine Included) (Jul 8, 2007),” “Glaxo to buy Sirtris, Maker of a Drug Based on Red Wine (Apr 8, 2008),” “Hoping Two Drugs Carry a Side Effect: Longer Life (Jul 22, 2008),” and “Quest for a Long Life Gains Scientific Respect (Sep 28, 2009).”

As problematic as *SIRT1* was as a proposed dominant longevity gene, the pharmacology around Sirt1 activators became entirely indefensible. Resveratrol was known to be not a direct Sirt1 activator^64,65^ and the effect of resveratrol on high fat-fed mice had been attributed to Sirt1 without any genetic validation^69^. A group at Sirtris and Harvard claimed direct Sirt1 activators with 1000 times the potency of resveratrol and biological activity in rodent models of diabesity^71^. Upon attempts to replicate the findings, none of these data were reproducible. In contrast, it was shown that SRT1720, SRT2183, SRT1460 and resveratrol do not activate Sirt1 with native peptide or full-length protein substrates but rather interact with the fluorophores used to assay sirtuins (Fig. 3), fail to lower blood glucose nor improve mitochondrial capacity in mice fed a high fat diet, and exhibit multiple off-target activities against diverse cellular targets^72^.

After GSK bought Sirtris for $720 million and undertook a multibillion dollar program to develop the proposed SRT compounds, there was a powerful driving force to rescue this program, which appeared to be chasing a fluorophore artifact and off-target effects^72,73^.

The proponents of resveratrol and SRT compounds as direct Sirt1 activators then published data claiming that the fluorigenic reporter interaction was mimetic of authentic aromatic interactions with Sirt1 substrates and further claimed that the ability of these compounds to activate Sirt1 depends on Glu230, an amino acid that is found in human, mouse and fly Sirt1 but—according to their own sequence alignment—not worm or yeast SIR2 homologs^74^. This report is troubling because the same senior author claimed that resveratrol was identified as an activator of yeast Sir2^58^ and worm *Sir-2.1*^66^. How can the original papers be correct and supported by new data if the specific interaction between resveratrol and Sir2 homologs depends on an amino acid not found in these enzymes? The defense of the resveratrol-Sirt1 interaction moved the goalposts so far that the authors took away their own first score. Without Glu230, resveratrol should not be able to activate Sir2^74^ but the senior author still claims that resveratrol activates Sir2 and, in his terms, gave the yeast cells the human equivalent of 50 extra years of life^46^. At what point will co-authors or the journals ask for one or both of the conflicting resveratrol papers^58,74^ to be retracted? Note that at the time of this writing, this group’s first misleading resveratrol Nature paper has >4500 citations^58^ and their misleading mouse Nature paper has 5000 citations^69^, making them not only the highest cited papers on resveratrol but apparently the highest cited original research publications in the entirety of the aging field, with thousands more citations than papers that have been foundationally important for replicable work that has produced deep insights into aging^35,36^.

We know that resveratrol and SRT compounds have specific binding interactions with fluorophores^64,65,72^, that the off-target effects of resveratrol are well documented^68,72,75^, and that when pterostilbene, a more bioavailable derivative of resveratrol was given to humans, it caused a dose-dependent increase in low density lipoprotein cholesterol^76^, which is contrary to expectations based on hitting Sirt1.

It is the view of this author that there are overwhelming data to reject resveratrol and SRT compounds as specific activators of Sirt1 and to ignore the concept of xenohormesis as it was never anything more than a story.

## *SIRT1* does Not Have the Properties Expected of a Mammalian Longevity Gene

There are roughly 20,000 genes in mammalian gene sets. Following the identification of loss of function alleles of *daf-2* as conferring longevity in worms^35,36^, loss of function alleles of several pituitary genes were shown to confer longevity in mice at the expense of size and fertility^37^. In principle, dominant longevity genes would be more exciting than loss of function alleles of growth genes because one could, in theory, generate mice of normal size and fertility that would be more fit for a longer time. Indeed, a popular book claims that sirtuin genes—identified as dominant longevity genes in yeast, conserved as such across all animals and activated by daily doses of resveratrol and nicotinamide mononucleotide (NMN)—make it such that we don’t have to age^46^.

Few scientists would have looked for longer-lived phenotypes in sirtuin overexpressing mice after seeing that invertebrate Sir2 overexpression doesn’t extend lifespan in clean worm and fly backgrounds^47^ or that loss of one copy of dSir2 extends lifespan in response to amino acid starvation^52^. However, mouse *Sirt1* transgenics were made after the initial nonreproducible invertebrate experiments were published^41,43^.

The first *Sirt1* transgenic model failed to show lifespan extension but showed lower blood cholesterol and blood glucose^77^. The paper was accompanied by a feature entitled “Is *Sirt1* a magic bullet for longevity?”^78^. Two additional groups made mice transgenic for *Sirt1* and showed that they are protected against some of the metabolic effects of high fat diet, also without extending lifespan^79,80^. Interestingly, mediation of metabolic responses to fasting were specifically examined and found to be unaffected in these mice^80^ but the paper was not written as a test of this foundational hypothesis of sirtuin research. In yet a fourth laboratory, *Sirt1* transgenic mice failed to show a longevity benefit but showed resistance to a liver carcinogenesis model driven in part by high fat diet^81^—this effect could potentially be due to the increased energy expenditure these mice exhibit on high fat^79,80^.

In a fifth laboratory, multiple brain-specific *Sirt1* overexpression mouse lines were constructed. Line 10 extended lifespan by 11% while line 1 did not extend lifespan at all. Rather than publish this as a negative result, the authors claimed that *Sirt1* transgenic line 10 revealed the inner workings of Sirt1 by virtue of higher relative overexpression of *Sirt1* mRNA in particular brain regions. The methods for this were not sound. In both transgenic lines examined, *Sirt1* mRNA was more highly overexpressed in dorsomedial hypothalamus (DMH) and lateral hypothalamus (LH), which they argued were important. With an n of 3 mice and showing no statistics, the investigators calculated that the overexpression fold-change was greater in these two regions than in the arcuate, essentially arguing that *Sirt1* will extend lifespan if it is more highly overexpressed in DMH and HL than arcuate^82^. Brain transgenic lines 2 through 9 have not been discussed, nor has a hypothalamic overexpresser tested the hypothesis that this effect was driven by *Sirt1* rather than a transgene insertion site or other artifact.

With respect to knockouts, a first report indicated that *Sirt1* knockout mice do not increase their physical activity in response to CR^83^ and another report claimed that Sirt1 is required for food anticipatory activity (FAA) by virtue of neuronal activities in the hypothalamus^84^. However, when *Sirt1* knockout mice were reexamined, it became apparent that these mice are constitutionally inactive on *ad libitim* feeding, have a higher oxygen consumption rate than wild-types, and are hyperphagic yet smaller than wild-types^85^. The lethargy of *Sirt1* knockout mice is a replicable result and, in fact, the activity difference is greater on high fat diet than on a standard chow diet, indicating that there is not a specific deficit in CR-induced activity^86^. The FAA-dependence on *Sirt1* could not be reproduced as neither whole body deletion, active-site substitution, forebrain deletion (*CamkIIα CRE)*, neuronal deletion (*Nestin* CRE), POMC neuron deletion (*Pomc* CRE) or tyrosine hydroxylase neuron deletion (*Th* CRE) of *Sirt1* eliminated FAA behavior^87^. The nonreplicability of the hypothalamic effects of Sirt1 is important because the same group which claimed that Sirt1 is required for FAA^84^ also claimed that specific ratios of *Sirt1* overexpression in hypothalamus versus arcuate are required to reveal a life-extending effect of this gene^82^.

In clinical research, best practices demand that hypotheses and primary endpoints are prespecified such that impartial observers will know when an intervention has failed to allow rejection of the null hypothesis. Indeed, the term HARKing was developed to warn scientists of the danger of forming Hypotheses After the Results are Known^88^. *Sirt1* was supposed to extend lifespan and it didn’t in four different laboratories^77,79–81^. Then it was supposed to extend lifespan when overexpressed in brain. When it did in one but not other transgenic lines, the authors found ways, *post hoc*, to weave a story around relative overexpression levels in regions of the brain^82^ based on behavioral characterization^84^ that also did not hold up to genetic analysis^87^. While HARKing can sometimes explain discoveries and it is certainly legitimate to test hypotheses that emerge from unanticipated results, it is illegitimate to simply construct a story around noisy data that move the goalposts to where the ball has fallen.

Human SNP data are now extensive and allow for discovery of rare or common variants that are associated with increased lifespan. SNPs in *SIRT1* have been interrogated and have not been shown to be associated with increased human lifespan^89,90^.

Carl Sagan taught us that extraordinary claims require extraordinary evidence. The thesis that what *SIR2* is doing for a CR yeast mother cell is so fundamentally conserved that we would see lifespan extension across invertebrates and vertebrates^18–20,44^ is not supported. While the line 10 brain-specific overexpresser has a longer lifespan^82^, it is difficult to reconcile this as strong positive support for the thesis because we have to stipulate that *SIR2* opposes longevity in the selected yeast model of CR-induced lifespan extension^15^, is dispensable in worms^47^, opposes the benefit in flies^52^, is dispensable for FAA experienced in CR^87^, and doesn’t generally extend lifespan in mice^77,79–81^ unless the transgene is confined to the brain and then depends either on insertion site, relative level of expression in particular brain regions, or innumerable other confounding effects^82^. The obvious conclusion is that while *SIR2* represses ERC formation in old mother yeast cells, it would have been amazing if this nonselected trait were to anticipate and prevent aging in animals—and it doesn’t.

Strangely, however, based on the lore from yeast, hyped predictions by industry, review articles and the popular press, negative results are rarely expressed as lack of support for *Sirt1* as a key mediator of the longevity benefit of CR. Indeed, the financial and intellectual investment in the idea that increased activity of the *Sirt1* gene would extend lifespan was so great that transgenics were made five times until this result could be scored positively^77,79–82^. Even then, the underlying data document clear evidence of nonreproducibility and HARKing^82^. Given the reproducibility problems documented in yeast^64^ and invertebrate^47,67^ aging assays and the bias toward publication of positive results^91^, one wonders how frequently any random transgene analyzed by five groups at multiple insertion sites would produce a report of increased lifespan.

## Six Other Sirtuins Have Been Extensively Searched for Longevity Phenotypes

It is frequently observed in human nature that the framing of questions has an influence on the answers that are given^92^. *SIR2* was first defined as a gene that does gene silencing^3,4^ and could also have been simply characterized as a founding member of NAD^+^-dependent deacylases^8–10^ with substrates and biological functions in different organisms that are poorly conserved and not part of a single vital process.

Longevity is such an age-old concern, however, that since the identification of the role of *SIR2* in old yeast mothers^14^, this gene has been compellingly framed as a dominant longevity gene that could extend lifespan by making an organism feel the beneficial stress of CR. Even when the effect of *SIR2*-related genes is precisely against the expectations of longevity^15,52^ or when no strong result has been obtained, an abundance of research groups have been willing to commit time and treasure to find lifespan-extending effects of sirtuins. Three types of biases are at work, namely framing bias^92^, confirmation bias^93^ and publication bias^91^.

The framing bias of sirtuin research is that review articles and the global lay literature defined sirtuins as longevity genes rather than genes conserved as protein lysine deacylases that were first identified as regulators of yeast gene silencing. This means that researchers would set out to find longevity phenotypes. Confirmation bias is that researchers who chose to work in this field thought these genes would positively confer longevity because that’s what they thought the yeast replicative aging assay^14^ and countless reviews taught. Moreover, the framing bias was established long before yeast and fly genetics indicated the exact opposite^15,52^. Imagine the research environment in which the first fungal *SIR2* longevity result was that *sir2* deletion produces an extraordinarily long lived yeast in CR^15^ and this has been conserved all the way to flies^52^. Framing and confirmation biases would then mobilize a community try to figure out why sirtuins are so bad for animal aging.

Positive results are easier to publish than negative results^91^. Many of the most positive-sounding longevity results were published in high profile journals despite a lack of rigor and were accompanied by breathless press coverage. In contrast, negative results are frequently abandoned by laboratories and, even when published, are frequently ignored in major review articles such that the general impression of working scientists can remain doggedly consistent with a false premise and a thoroughly failed thesis. At the time of this writing, for example, the Proceedings of the National Academia of Sciences paper claiming that dSir2 mediates the longevity benefit of CR has more than 1500 citations in Google Scholar including hundreds of citations by highly cited review articles^43^, while the Genome paper showing that loss of a copy of the dSir2 is required for lifespan extension in flies with amino acid restriction has only 13 citations and has yet to be cited by a major review article^52^.

The asymmetries between publication ease and rewards of positive versus negative data have been magnified by commercial and reputational interests that are deeply tied to tested and disproven theories. A prime example of this is the perseverance to find a mechanism by which resveratrol activates human Sirt1 even when the mechanism proposed^74^ is fully inconsistent with the same author’s claims that compound was discovered as a Sir2-stimulating yeast lifespan extender^58^.

In the light of the global search for prolongevity functions of sirtuins, the set of potential mediators has been expanded. Though *SIRT1* was said to be the homolog of *SIR2*, it was proposed that all or any of the seven sirtuins could be dominant longevity genes^18–20,44^. Here we consider the functions of *SIRT3* and *SIRT6*—interesting enzymes to be sure—but not conserved as longevity genes.

The mitochondrial deacetylase Sirt3 was claimed to be a specific activator of succinate dehydrogenase^94^, long chain acyl coA dehydrogenase^95^, superoxide dismutase^96^ and other enzymes. This doesn’t entirely make sense because deacetylases do not specify where acetyl groups are put on, and it was pointed out that the same lysines—generally found at enzyme active sites with perturbed *p*K_a_ values—are modified by multiple acyl groups by mass action in disparate conditions of metabolic stress^97,98^. Seen in this light, Sirt3 is less a specific enzyme activator than a constitutive remover of acetyl modifications that are deposited due to the chemical reactivity of Lys residues with the partial positive charge on the carbonyl C of acetyl coA^97^. *Sirt3* transgenic mice are not longer-lived but are resistant to inducers of mitochondrial stress such as doxorubicin that produce mitochondrial hyperacetylation^99^. Remarkably, even before the *Sirt3* transgenic was investigated, a review article on Sirt3 was entitled “Forever young: SIRT3, a shield against mitochondrial meltdown, aging, and neurodegeneration”^100^.

*Sirt6* knockout mice are small, lack subcutaneous fat, and are short-lived with a deficiency in DNA repair. The title of the paper termed this an “aging-like phenotype” but it looks more like a failure to thrive on top of low blood sugar, colitis and low circulating IGF-1^101^. *Sirt6* transgenic mice on a mixed background had a 10–15% lifespan extension in males but not females^102^. Though the male-specific longevity effect correlated with depressed IGF-1 circulation, the *Sirt6* knockout also had low IGF-1, such that the mechanism of lifespan extension was not clear^101^. The same group has continued to generate transgenics in additional mouse backgrounds and has now shown that on a C57BL/6JOlaHsd mouse background, *Sirt6* overexpressers remain more active and have extended lifespan in both sexes with gene expression programs, depressed IGF-1 circulation and metabolic characteristics that are generally more youthful^103^. In discussing the strain-specificity of these results, the authors noted that female mice circulate lower levels of IGF-1, such that if the mechanism of lifespan extension by Sirt6 depends on depressed IGF-1, the effect of Sirt6 would be dampened in strains and sexes with lower IGF-1^103^. However, we know that Sirt6 cannot be a negative regulator of IGF-1 circulation because *Sirt6* knockout mice have low circulating IGF-1^101^.

A consistent result from *Sirt6* knockouts and transgenics is that Sirt6 is a positive regulator of gluconeogenesis, which appears to benefit older mice^101,103^. However, one would expect the same mechanism to be contraindicated for humans because higher blood glucose tracks with better health in old mice and with worse health in people^104^. Indeed, metformin has been contemplated as a longevity medicine^105^ that works, in part, by depressing gluconeogenesis^106^.

Those who are working on sirtuins with respect to aging are surely pleased if not relieved that some mouse strain backgrounds and circumstances have been found in support of some aspects of the sirtuin-aging thesis. However, the results fall far short of support the overall thesis that sirtuins are conserved as longevity genes because the premise was already falsified in yeast and invertebrates. Moreover, if seven sirtuin genes were indeed conserved to promote animal lifespan, positive results would be penetrant across mouse strains and sexes, such as they are with inactivating mutations in growth hormone signaling^37^. Here it is important to note that some of the dwarf mutant genes were identified in “mouse fancier” backgrounds before being crossed into standard laboratory strains^107^. This caused no problem whatsoever because pituitary pathway genes clearly regulate aging in both sexes in all strains examined. It doesn’t matter which laboratory examines them and we don’t have to get into handwaving about insertion sites or make *post hoc* arguments about relative expression in beneficial versus nonbeneficial tissues.

In contrast, examination of mammalian sirtuin data reveal example after example in which framing bias, confirmation bias and publication bias perturbed the characterization of interesting results about physical activity, mitochondrial housekeeping, DNA repair and gluconeogenesis as though they primarily reflected insights into underlying processes of aging that are so fundamental they could have been anticipated by yeast cell biology.

In this researcher’s view, though the genes are interesting, there is no specific insight about aging that has emerged from the analysis of seven sirtuin genes in mammals. This researcher continues to read in reviews and seminar introductions that sirtuins mediate the longevity effects of CR but this is not true in yeast^15,17^, not true in invertebrates^47,52^, and not true in mice^87^. Indeed, investigators might better understand the function of these genes if they were unshackled from the falsely premised framing bias that sirtuins are conserved longevity genes.

## There is Little Evidence that the Benefits of Repletion of the NAD^+^ Metabolome is Primarily Mediated by Sirtuins

Shortly after discovery of the vitamin activity of nicotinamide riboside (NR)^108^, we used the yeast system to show that NR increased cellular NAD^+^, Sir2 activity and replicative lifespan^109^. We did not claim that provision of NR would extend human lifespan by virtue of increasing the activity of sirtuins. Though yeast replicative aging is an assay one can employ in the laboratory, it struck us as fantastical and a violation of Rule 2 that rare, old yeast mothers employed a nonselected genetic trait that would anticipate the causes of human aging.

During this era, the effect of resveratrol on high fat-fed mice had been described with the previously discussed lack of evidence of mediation by Sirt1^69^. In the course of characterizing the mechanism of action of resveratrol in mice, it was discovered that AMP kinase is the apparent direct target, but a role for Sirt1 had not been discarded^75^. Thus, when overfed mice were shown to have improved metabolism when fed NR^110^, the investigators did not probe the mechanism genetically but rather decided to blot for the acetylation state of nuclear transcription factor Foxo1 and mitochondrial Sod2. Seeing lower levels of acetylated Foxo1 and Sod2, they claimed that the beneficial effects of NR were mediated by Sirt1 and Sirt3^110^. As with the unsubstantiated claim that resveratrol activates Sirt1 to deacetylate PGC1α^69^, the investigators did not look at what was causing these proteins to be acetylated, whether Sirt isozymes were really responsible for their deacetylation, or whether these modifications were important to the mechanism of action of NR.

Similarly, when mice were dosed with NMN, it was claimed that NMN suppressed age-associated body weight gain, enhanced energy metabolism, promoted physical activity and insulin sensitivity. No genetic evaluation was done of the mediators but the discussion speculated on activation of SIRT1, SIRT3, SIRT4, SIRT5 and SIRT6^111^.

The redox functions of NAD coenzymes, NAD^+^, NADH, NADP^+^ and NADPH, are essential for fuel oxidation, oxidative phosphorylation, gluconeogenesis, ketogenesis, nucleotide, lipid and steroid synthesis, and detoxification of reactive oxygen species^112^. These coenzymes function as the central catalysts of metabolism, the set of all of the processes that allow us to convert everything we eat into everything we are and everything we do.

For reasons that are hard to understand, the vital redox functions conferred by the NAD system have been nearly ignored throughout much of the sirtuin era. Perhaps because the sirtuin field embraced the fantastical idea that a yeast gene anticipated and could prevent the causes of aging in animals, speculative mechanisms related to NAD boosting often invoke the idea that supraphysiological levels of NAD^+^ would get sirtuins to levels of activity they were capable of in youth and/or in a leaner environment. These ideas persist without genetic testing.

Quantitative targeted NAD metabolomics^113^ has revealed that the NAD system is functionally disturbed by conditions of metabolic stress including fatty liver^114^, peripheral^115^ and central^116^ neurodegeneration, noise-induced hearing loss^117^, DNA damage^118^, heart failure^119^, postpartum^120^, activation of specific oncogenes^121^, alcoholic liver disease^122^, interleukin 8 signaling^123^, viral infection^124^, mitochondrial disease^125^, and inflammaging^126^. Thus, when we have applied NR as a potential remedy, we have done so in the context that there is a functional deficit in key metabolites such as NAD^+^ or NADPH that are putting cells and tissues at risk of not meeting bioenergetic needs, not being able to repair DNA, not being able to detoxify reactive oxygen species, not being able to conduct anabolic processes, not being able to support the activity of a monoADPribosylating PARP family member that is specifically induced, etc. While many redox and repair mechanisms associated with repletion of the NAD system are pleiotropic, we urge researchers to use genetic and other sound analytical techniques to probe NAD repletion mechanisms rather than just doing a western blot and weakly asserting SIRT-dependent mechanisms. Reviewers and editors should expect more as well.

## Conclusions

The NAD system features enzymes such as PARP1 that are intensely activated by the appearance of DNA damage^118^, monoADPribosylating PARPs that are strikingly induced by the innate immune system^124^, SARM1 that is activated by accumulating NMN^127^, CD38 that is activated by interleukin 8^123^, and vital redox enzymes that are controlled by metabolites and electron fluxes. While sirtuins carry out interesting biochemical reactions and perform important functions for organisms, their degree of regulation is modest compared to most other enzymes that function in the NAD system. In addition, the degree of hype, framing and nonreproducibility in the sirtuin literature is persistently misleading. Dispassionate analysis of their functions does not support assignment as longevity enzymes or as principal mediators of the effects of NAD repletion.
